# Supplementary material for: Predictive effect of investor sentiment on current and future returns in emerging equity markets
Source: PLoS One. 2023 May 18;18(5):e0281523. doi: 10.1371/journal.pone.0281523 (PMC10194879; doi:10.1371/journal.pone.0281523)
Supplement: Attachment — Submitted filename: Response to Reviewer.docx [file pone.0281523.s001.docx]

**Author Response Letter**

**Dear Editor and Reviewers**

**First, thanks to all of you for reviewing the research paper and for your valuable suggestions. All the suggestions have been incorporated, which improved the document. The revised paper is being submitted with a request to kindly consider the same for publication.**

**The suggested changes have been incorporated with detail as follows:**

**Reviewer 1**

**First, thank you for reviewing the research paper and for your valuable suggestions. All the suggestions have been incorporated, which improved the document. The revised paper is being submitted with a request to kindly consider the same for publication.**

**The suggested changes have been incorporated with detail as follows:**

**Comments to the Author:** The authors should better highlight the research gap and motivation of the study.

**Author Response:** The Research Gap is revised in the manuscript as per the suggestions and highlighted in the document.

**Comment to the Author:** The contribution of the papers should be clearly explained.

**Author Response:** The contribution of the papers is clearly explained in the last section of the manuscript.

**Comment to the Author:** The literature review section should discuss a few latest papers on the topic.

**Author Response:** Change has been incorporated into the document (The latest studies such as Wang et al. (2021) added to the document).

**Comment to the Author:** The choice and rationale to justify the use of specific methods needs to be improved.

**Author Response:** The choice and rationale is revised and improved by justifying the use of specific methods.

**Comment to the Author:** The discussion on the results requires to be expanded, where the focus should be paid to corroborating the findings with previous evidence, and economic interpretation of the results should be improved.

**Author Response:** The discussion on the results is expanded by supporting the findings, and the economic interpretation of the results is also improved.

**Reviewer 2**

**First, thank you for reviewing the research paper and for your valuable suggestions. All the suggestions have been incorporated, which improved the document. The revised paper is being submitted with a request to kindly consider the same for publication.**

**The suggested changes have been incorporated with detail as follows:**

**Comment to the Author:** I can see that there is a contribution of the study but is not well-written or explicitly states what novelty is being added to the study.

**Author Response:** The contribution of the study is revised to explicitly explain how ‘novelty’ is being added to the study.

**Comment to the Author:** What is the rationale behind using underlying methods in the current research? Why the authors did not employ other similar techniques and preferred this technique solely?

**Author Response:** The choice and rationale of the underlying method is revised and improved by justifying the use of a specific technique.

**Comment to the Author:** Are there any other studies on a similar methodology? If yes, please cite them to have a better empirical justification.

**Author Response:** yes, there are several studies available on a similar methodology, in text citations and references are inserted at the appropriate place.

**Comment to the Author:** What are the future prospects of this study? Adding the future directions along with the implications for the individual market would be the greater advantage of the study.

**Author Response:** The Future Prospects, Future Directions, and Implications in the conclusion and recommendation section have been added.

**Comment to the Author:** I found some grammatical and typo errors in the manuscript. In that case, a professional proofreader can polish and enhance the quality of your manuscript.

**Author Response:** The document is checked properly through grammar. All typo errors have been removed and all suggested changes in the entire manuscript are done.

**Regards**

Authors
